# (M)Unc13s in Active Zone Diversity: A *Drosophila* Perspective

**DOI:** 10.3389/fnsyn.2021.798204

**Published:** 2022-01-03

**Authors:** Chengji Piao, Stephan J. Sigrist

**Affiliations:** ^1^Institute for Biology/Genetics, Freie Universität Berlin, Berlin, Germany; ^2^NeuroCure Cluster of Excellence, Charité Universitätsmedizin, Berlin, Germany

**Keywords:** active zone diversity, pre-synaptic plasticity, active zone ultrastructure, release sites, synaptic transmission

## Abstract

The so-called active zones at pre-synaptic terminals are the ultimate filtering devices, which couple between action potential frequency and shape, and the information transferred to the post-synaptic neurons, finally tuning behaviors. Within active zones, the release of the synaptic vesicle operates from specialized “release sites.” The (M)Unc13 class of proteins is meant to define release sites topologically and biochemically, and diversity between Unc13-type release factor isoforms is suspected to steer diversity at active zones. The two major Unc13-type isoforms, namely, Unc13A and Unc13B, have recently been described from the molecular to the behavioral level, exploiting *Drosophila* being uniquely suited to causally link between these levels. The exact nanoscale distribution of voltage-gated Ca^2+^ channels relative to release sites (“coupling”) at pre-synaptic active zones fundamentally steers the release of the synaptic vesicle. Unc13A and B were found to be either tightly or loosely coupled across *Drosophila* synapses. In this review, we reported recent findings on diverse aspects of *Drosophila* Unc13A and B, importantly, their nano-topological distribution at active zones and their roles in release site generation, active zone assembly, and pre-synaptic homeostatic plasticity. We compared their stoichiometric composition at different synapse types, reviewing the correlation between nanoscale distribution of these two isoforms and release physiology and, finally, discuss how isoform-specific release components might drive the functional heterogeneity of synapses and encode discrete behavior.

## Introduction

Upon the arrival of action potentials at the pre-synaptic terminal, neurotransmitters stored at synaptic vesicles (SVs) are released from a specialized region of the plasma membrane called active zones (AZs). Although AZs largely share the same complement of proteins, the probability of SV release varies tremendously across different neurons and even between AZs formed by the same neuron (Akbergenova et al., 2018), resulting, *inter alia*, in differences in the short-term plasticity of SV release. While short-term facilitation is typical for synapses with a high initial release probability, short-term depression is often observed at ones with a low initial release probability (Fioravante and Regehr, 2011). Importantly, synapses with different adaptive behaviors might convey signals in defined frequency ranges.

Aspects of functional diversity have previously been attributed to various AZ sizes and shapes (Atwood and Karunanithi, 2002; Moser et al., 2020; Wichmann and Kuner, 2022). The number, density, and distribution of Ca^2+^ channels in or near the AZ area account for AZ heterogeneity as well (Holderith et al., 2012; Eltes et al., 2017). The functional differentiation of AZs can also be achieved by building synapses from distinct molecules or isoforms or varied quantities, densities, and nanoscale arrangements of the very same molecule (Nusser, 2018; Karlocai et al., 2021). The Unc13 protein family is of particular relevance here. A recent enlightening review by Jeremy Dittman highlighted Unc13s as the hubs of AZs, coordinating diverse aspects of synaptic transmission (Dittman, 2019). The roles of Unc13 are fulfilled by different isoforms. Mammals contain three brain-specific Munc13 isoforms, namely, Munc13-1, bMunc13-2, and Munc13-3. Munc13-1 is the dominant isoform expressed throughout the brain, while Munc13-2 and Munc13-3 display strikingly distinct expression patterns. Munc13-2 is preferentially present in the rostral brain regions, and Munc13-3 is almost exclusively present in the cerebellum of rats (Augustin et al., 1999). Synapses employing different Unc13 isoforms as priming factors exhibit different forms of short-term plasticity (Rosenmund et al., 2002). However, the neonatal lethality of Munc13-1/2 double mutants makes it difficult to decipher how their distinct release attributes contribute differently to information coding and behavior. Luckily, recent studies of two Unc13 isoforms in *Drosophila* have linked their molecular functions to discrete behavioral components (Table 1), making the protein a great candidate to unveil the design principles tuning functional diversity over a spectrum of synapse types.

**Table 1:** Unc13A vs. Unc13B in Ca^2+^ channel coupling distance and functions in *Drosophila*.

	**Synapse type**	**Unc13A**	**Unc13B**	**Method**	**References**
Average	NMJ	70 nm	120 nm	STED	Bohme et al., 2016
distance to Ca^2+^		76.8 nm	145 nm	Simulation	
channel/AZ center	ORN-derived AZs at AL	83 ± 6 nm	115 ± 8 nm	STED	Fulterer et al., 2018
	LN-derived AZs at AL	74 ± 2 nm	102 ± 3 nm		
	PN-to-KC	74 ± 4 nm	102 ± 1 nm		
	ePN-to-LHN	61 ± 1 nm	101 ± 4 nm	gSTED	Pooryasin et al., 2021
	iPN-to-LHN	60 ± 2 nm	102 ± 4 nm		
	KC-derived AZs at MB lobes	100–150 nm	150–250 nm		Woitkuhn et al., 2020
SV release	NMJ	eEJC amplitude ↓*↓↓* mEJC amplitude ↑ mEJC frequency ↑ PPR ↑ Time to peak ↑	eEJC amplitude ↓ mEJC amplitude ↔ mEJC frequency ↔ PPR ↔ (L3 stage)/↑ (L2 stage) Time to peak ↔	Two-electrode voltage clamp Current clamp	Bohme et al., 2016; Ramesh et al., 2021
	ORN-to-PN	EPSC amplitude ↓ PPR ↑ Time to peak ↑	EPSC amplitude ↔ (↓) PPR ↔ Time to peak ↔	*In vivo* whole-cell patch clamp	Fulterer et al., 2018
	LN-to-LN	Peak amplitude ↔ Time to peak ↔	Peak amplitude ↓ Time to peak ↔		
	KC-to-MBON γ1pedc> α/β	EPSC amplitude ↓ Short-term depression ↓	/		Woitkuhn et al., 2020
	ePN-to-KC	MaxΔF/F_0_ ↓ Time to peak ↑	MaxΔF/F_0_ ↓ Time to peak ↔	*In vivo* calcium imaging	Pooryasin et al., 2021
		MaxΔF/F_0_ ↓↓ Time to peak ↑			
Innate behavior	Heterozygous mutants	Ethanol preference ↑		CAFÉ assay	Das et al., 2013
		Ethanol sensitivity ↓		Ethanol LOR assay	Xu et al., 2018
	Kenyon cell	Odor avoidance ↔		T-maze	Bohme et al., 2019; Woitkuhn et al., 2020
		Shock avoidance ↔			
	ePN	Odor attraction ↓	Odor attraction ↑		Pooryasin et al., 2021
		Odor avoidance ↓	Odor avoidance ↓		
	iPN	Odor attraction ↔	Odor attraction ↔		
		Odor avoidance ↔	Odor avoidance ↔		
Memory	Kenyon cell	STM ↓↓	STM ↓		Bohme et al., 2019; Woitkuhn et al., 2020
		MTM ↓↓	MTM ↓		
		ASM ↓↓	ASM↔		
		ARM ↓	ARM↔		

*AL, antennal lobe; ARM, anesthesia-resistant memory; ASM, anesthesia-sensitive memory; AZ, active zone; eEJC, evoked excitatory junction current; ePN, excitatory projection neuron; EPSC, excitatory postsynaptic current; gSTED, time-gated super-resolution stimulated emission depletion microscopy; iPN, inhibitory projection neuron; KC, Kenyon cell; LHN, lateral horn neuron; LN, local interneuron; MB, mushroom body; MBON, mushroom body output neuron; mEJC, miniature excitatory junction current; MTM, mid-term memory; NMJ, neuromuscular junction; ORN, olfactory receptor neuron; PN, projection neuron; PPR, paired pulse ratio; STED, super-resolution-stimulated emission depletion microscopy; STM, short-term memory*.

## The Fly Unc13 Isforms: Unc13A vs. Unc13B

**Figure 1:**
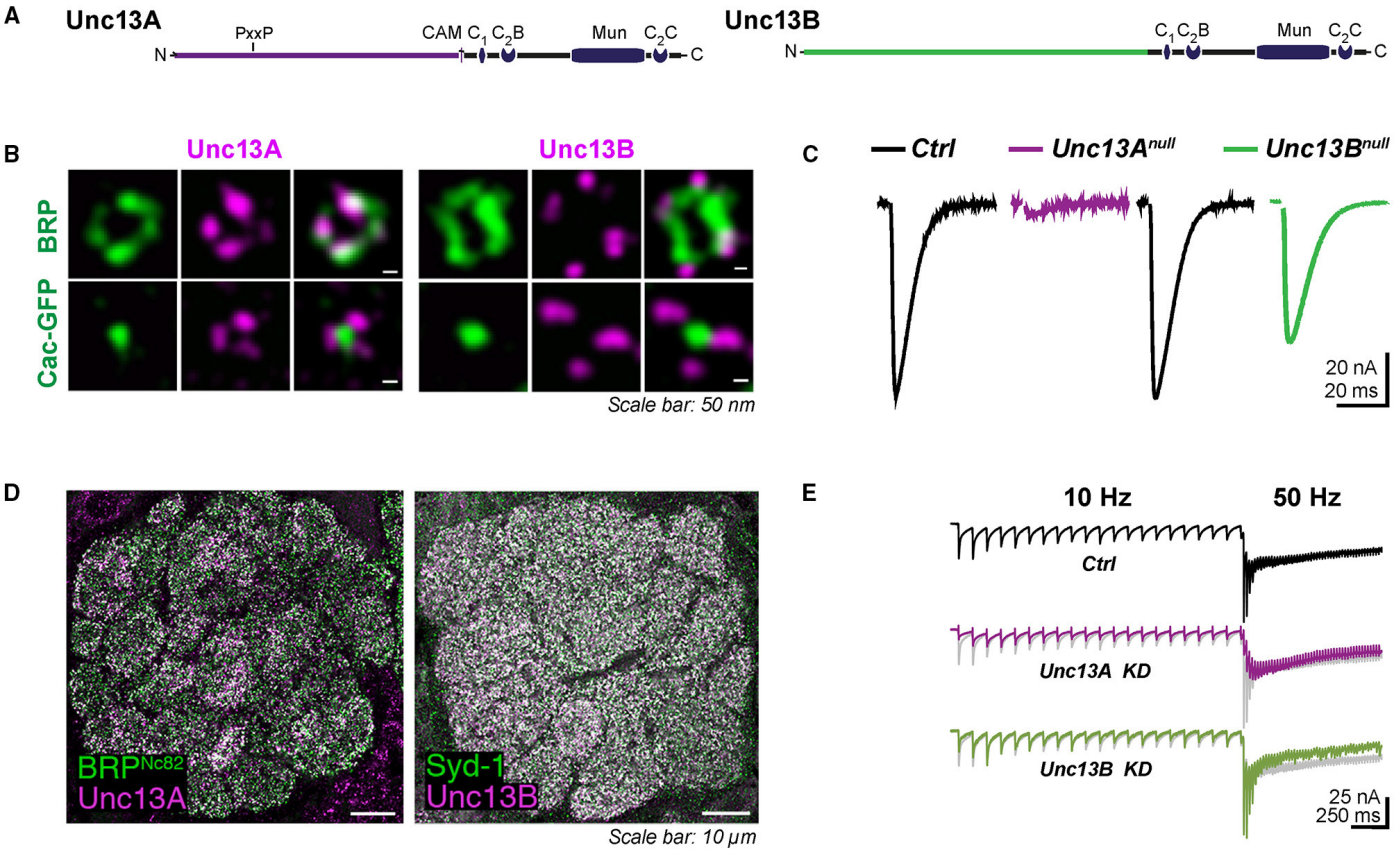
**(A)** Domain structures of Unc13A and Unc13B. **(B)** Nano-topology of Unc13A and B at active zone (AZ) and their coupling distance to Ca^2+^ channels at *Drosophila* neuromuscular junctions (NMJs) shown by super-resolution-stimulated emission depletion microscopy (STED) images. Synaptic transmission at NMJ is dominantly controlled by Unc13A. Null mutants of *Unc13B* only showed a mild reduction in the response evoked, recording traces shown in **(C)**. **(D)** Expression pattern of Unc13A and Unc13B in the antennal lobe of *Drosophila*. **(E)** Synaptic currents evoked recorded at fast-depressed olfactory receptor neuron (ORN)-to-projection neuron (PN) synapses. **(A–C)** are modified from Bohme et al. (2016) and **(D,E)** are modified from Fulterer et al. (2018).

The (only) fly *Unc13* locus was identified in 1999 (Aravamudan et al., 1999). In line with the function of its *Caenorhabditis elegans* and mammalian homologs, characterizing a *Drosophila unc13* null mutant revealed its essential role for SV exocytosis *per se*. The locus generates two isoforms, namely, Unc13A and Unc13B, which differ in their extended N-termini. The identical C-terminal region encompasses a C1 domain, a regulatory C2B domain, followed by a large catalytic Munc homology (MUN) domain and a C2C domain (Figure 1A). This arrangement shared by the isoforms is highly conserved across evolution, suggesting conserved functions of the C-terminal fragment in SV priming and molecularly defining vesicle release sites. Reconstitution data suggest that the entire C1-C2B-MUN-C2C complex can bridge the plasma and vesicle membranes; the very C-terminal part C2C domain is thought to be critical for this ability (Liu et al., 2016; Quade et al., 2019). The central extent of the homologous region, known as the MUN domain (Xu et al., 2017), is key for the Unc13 SV priming function and is the minimal Unc13 protein domain required to carry out the priming activity (Basu et al., 2005). However, how the MUN domain is involved in the SNARE assembly exactly remains largely elusive. It has been suggested that its weak interaction with the SNARE syntaxin-1 catalyzes the transition of closed syntaxin-1 to an open conformation, thus enabling the SNARE assembly (Ma et al., 2011; Magdziarek et al., 2020). In mammals, a member of the extended (M)Unc13 family, CAPS, shares only the Unc13 MUN domain (Koch et al., 2000) and is essential for dense-core vesicle exocytosis (Berwin et al., 1998; Rupnik et al., 2000; Liu et al., 2008). A CAPS homolog with a related function is also found in *Drosophila* (Renden et al., 2001). The MUN domain may also interact with synaptobrevin-2 and recruit vesicles. Other recent studies have proposed a role of the (M)unc13-1 MUN domain cooperating with (M)unc18-1 in orchestrating the SNARE assembly by stabilizing the template complex formed by Unc18, syntaxin-1, and synaptobrevin-2 (Wang et al., 2019; Shu et al., 2020). The C2B domain, which can bind to Ca^2+^ and phosphatidylinositolphosphate, likely acts here as a release probability and short-term plasticity modulator (Shin et al., 2010); the C1 domain, positioned next to the C2B domain, is meant to regulate diacylglycerol-dependent release augmentation (Basu et al., 2007). In addition to these two signaling domains, Unc13A harbors a calmodulin-binding sequence (CAM) that has been shown to be essential for regulating the priming activity and short-term plasticity (Lipstein et al., 2012, 2013; Piotrowski et al., 2020), whereas it does not exist in the N-terminal part of the B isoform (Xu et al., 1998). The N-terminus of Unc13A also comprises a proline-rich motif (PxxP) binding to the SH3 domains of Rim-binding protein *in vitro* (Bohme et al., 2016). The very similar domain structure mode is shared by all mammalian Unc13 members: Munc13-1, ubMunc13-2, bMunc13-2 and Munc13-3, and Unc13-L and Unc13-S in *C. elegans*. The canonical C-terminal CAM-C1-C2B-MUN-C2C complex presents in all Munc13 isoforms, and the N-terminal regions of Munc13-1 and ubMunc13-2 contain an additional C2A domain, which is absent in fly. The calmodulin-interacting motif and C2A domain also exist in nematode Unc13-L.

## Distinct Nanoscopic Distributions of Unc13A and Unc13B in Active Zones

To couple the SV release to electrical stimulation by action potentials, calcium ions entering the cell through voltage-gated Ca^2+^ channels activate the Ca^2+^ sensor synaptotagmin on the SVs to trigger fusion. The efficacy of synaptic transmission depends largely on the distance between SVs and voltage-gated Ca^2+^ channels. The two major fly isoforms of Unc13, Unc13A, and Unc13B are found co-expressed within individual AZs but astonishingly with different nanoscopic patterns. The nano-topology was first mapped at the peripheral neuromuscular junctions (NMJs) of *Drosophila* larvae using dual-color super-resolution-stimulated emission depletion (STED) microscopy (Bohme et al., 2016). The pre-synaptic voltage-gated Ca^2+^ channel α 1 subunit encoded by *Cacophony* forms a cluster in the center of AZ labeled by Bruchpilot (BRP) in *Drosophila* (Kawasaki et al., 2004; Fouquet et al., 2009). Unc13A was localized closer to the Cacophony cluster. Unc13B, in contrast, was found at a greater distance (Figure 1B). The average distances from the AZ center defined by the center of the BRP signal (overlaying the AZ-central Cacophony cluster) for Unc13A and Unc13B were measured to be about 70 nm and 120 nm at NMJ, respectively. This “design rule” of distinctly patterned nanodomain spacing of the two components relative to the Ca^2+^ channels was subsequently found to apply to all synapses studied so far in the central nervous system (CNS) of *Drosophila*. Concretely, the A isoform was found at 74–83 nm from the center of AZ, while the B isoform was, with a distance of 102–115 nm, at different relay synapses within the *Drosophila* olfactory system, including olfactory receptor neuron (ORN), projection neuron (PN), and Kenyon cell (KC) output synapses (Fulterer et al., 2018; Woitkuhn et al., 2020). Moreover, this principle of Ca^2+^ channel coupling distance for the two isoforms applies to not only excitatory but also inhibitory synapses. A three-channel two-dimensional time-gated STED (gSTED) imaging on PN-derived AZs in the lateral horn (LH) showed that Unc13A localized at about 60 nm relative to the AZ center, and the B isoform spots were still found at further distances at inhibitory PN (iPN) output synapses. These were the same as the coupling distances at the terminals of cholinergic excitatory PN (ePN) in the LH (Pooryasin et al., 2021).

Which molecular scenario might underlie this astonishing degree of release site nanoscale patterning? In addition to the release sites proper, an evolutionarily conserved set of AZ scaffold proteins has been suggested to function in the spatial organization of synapse nano-topology, including RIM, Rim-binding protein (RimBP), Liprin-α, and the glutamate (E), leucine (L), lysine (K), and serine (S) rich protein (ELKS)-family BRP. The Unc13A/B nano-topology design present across *Drosophila* synapses is seemingly tuned by two clusters of AZ scaffold proteins, namely, BRP/RimBP and Syd-1/Liprin-α complex. Syd-1 and Liprin-α, according to intravital live imaging, arrive early at nascent AZs and initiate the AZ assembly (Owald et al., 2010). Together with this complex, Unc13B clusters appear before the emergence of Unc13A along the AZ maturation trajectory and, finally (after synapse maturation is completed), colocalize with them at the outer edge of matured AZs at NMJ. Syd-1 and Liprin-α may recruit Unc13B by interacting with its N-terminus, which normally plays a role in the localization of Unc13 protein. The Unc13B accumulation at NMJ AZs depends genetically on the Syd-1/Liprin-α complex. Its expression was dramatically reduced at the NMJs of *liprin-*α and *syd-1* mutants, whereas *brp* knockdown or *Rimbp* deletion had no effect on Unc13B levels. In contrast, the A isoform is recruited via the AZ scaffold BRP/RimBP complex. The N-terminus is essential here for the AZ anchoring of Unc13A and interacts directly with the SH3 domain of RimBP and the N-terminal region of BRP (Bohme et al., 2016). Unc13A levels at both the larval NMJ and adult brain of *Drosophila* are tightly regulated by BRP levels. An additional gene copy number of BRP drives the upscaling of Unc13A, whereas pan-neural *brp* knockdown results in the almost complete loss of Unc13A (Huang et al., 2020; Huang and Sigrist, 2021).

Importantly, though both isoforms in the *Drosophila* brain are expressed in all neuropile areas, their relative abundance varies greatly among different types of synapses. Within the antennal lobe, the first processing center of the fly olfactory system, ORN-derived AZs display a high release probability (Kazama and Wilson, 2008) and are enriched for Unc13A, shown by STED to be tightly coupled to the Ca^2+^ channels. In contrast, the slow and facilitating local interneuron (LN) output synapses appear as Unc13B-dominated (Figure 1D). The second-order neurons of the olfactory circuit, called PNs, convey information from the antennal lobes to both the mushroom body calyx and LH. The PN terminals synapse onto KC dendrites in the calyx and PN-output synapses here are strongly enriched for Unc13B (Fulterer et al., 2018). Interestingly, the ratio of Unc13A/B in the higher memory center, known as the mushroom body, in turn, shows differences between specific bundles of axons (“lobes”) of mushroom body intrinsic neurons. The A isoform here is enriched in the two prime (α′ and β′) lobes and the γ lobe, while Unc13B exhibits higher levels in the α and β lobes (Woitkuhn et al., 2020). Additionally, the two isoforms are recruited to the AZ via the AZ master proteins BRP and Syd-1, respectively, across the synapses of the *Drosophila* brain (Fulterer et al., 2018; Huang et al., 2020). Hereby, the relative levels of two Unc13 isoforms at different synapses correlate strongly with the BRP/Syd-1 ratio. The AZs rich in Unc13A or B show likely contrasting release characteristics, which will be discussed later. The molecular abundance differences of two isoforms across the CNS probably produce functional differentiation, which is important for the information transfer within neuron networks.

## Unc13A and Unc13B Control Phasic vs. Tonic Release Across *Drosophila* Brain Synapses

As has been said above, analyzing the two isoforms unmasked drastic differences in their nanoscopic distribution at the level of the individual AZ. Subsequent work across several synapses showed consistently that the two isoforms, as suggested by their either tight or loose nanoscale coupling to the central Ca^2+^ channel cluster, promote distinct release features concerning short-term synaptic plasticity: Unc13A mediates fast phasic and Unc13B slow tonic release. We sketch these findings in the following.

Synaptic transmission at the larval NMJ seems to be dominantly controlled by Unc13A, as Unc13A deletion at NMJs decreased the evoked neurotransmitter release by almost 90% (Figure 1C) and greatly reduced the number of docked SVs close to the Ca^2+^ source (Bohme et al., 2016). On the single AZ level, the evoked SV release correlated strongly with the local BRP and Unc13A level (Peled et al., 2014; Reddy-Alla et al., 2017). The relation between evoked release and BRP was shown to be mediated by Unc13A, which, in turn, is stoichiometrically recruited by BRP. Indeed, the discrete clusters of Unc13A within individual AZs, at NMJ AZs typically 4–5, might well represent discrete SV release sites (Reddy-Alla et al., 2017). Here, the functional aspect of Unc13A generating “release sites” is obviously executed by the conserved C-terminal part of Unc13A, while the topological aspect of stable anchoring in defined nanoscopic positions depends on the Unc13A N-terminus. Expressing only the C-terminal part of it generates release sites at atypical locations (Reddy-Alla et al., 2017). This concept of Unc13 clusters forming release sites was independently described for central mammalian synapses (Sakamoto et al., 2018).

Tight and loose coupling to the Ca^2+^ channels of the two isoforms, sharing the very same C-terminal part, is thus associated with distinct release features and short-term synaptic plasticity, as best described at the first relay synapses of the fly olfactory circuit due to ideal electrophysiology recording conditions here. These ORN-to-PN synapses are cholinergic. Notably, in terms of post-synaptic signal reception, two components with distinct signaling kinetics were described pharmacologically at ORN-to-PN synapses, as a fast and a slow excitatory post-synaptic current (EPSC) component could be isolated when applying the nicotinic agonist imidacloprid or curare. The fast curare-sensitive component depressed more rapidly than the imidacloprid-sensitive component, presumably mediated by distinct types of acetylcholine receptors or distinct states of the same receptor (Nagel et al., 2015; Nagel and Wilson, 2016). As has been mentioned above, when knocking down the dominant Unc13A isoform at the ORN terminals, the fast, phasic response recorded at the PN was almost absent, indicated by a drastic reduction in peak amplitude and a delay in the time to peak. By contrast, Unc13B has a rather modest contribution to the signal transduction at ORN-to-PN synapses here, probably contributing to the tonic release component (Figure 1E). Taken together, these findings suggest the attractive idea that a specific release component, mediated by either Unc13A or B, might be “matched” to a specific post-synaptic neurotransmitter receptor, resulting in the establishment of two parallel “information channels” across one synapse.

The ORN terminals exhibit high release probability and feature a robust short-term depression upon repetitive stimulation. The loss of Unc13A at ORN-derived AZs results in a drastic shift toward short-term facilitation, suggesting that Unc13A dominates the SV release probability here (Fulterer et al., 2018). In line with this, transmission and short-term synaptic plasticity at KC-to-mushroom body output neuron γ1pedc> α/β synapses operate with a high SV release probability, which is promoted by Unc13A as well (Woitkuhn et al., 2020).

Although the Unc13B isoform seems to be somewhat dispensable at these synapses with a high release probability, it is clearly not just a functionally redundant “minor” member of the Unc13 family in the *Drosophila* CNS. As has been mentioned previously, the distribution of Unc13B is rather homogeneous over the antennal lobes of the fly brain, which matches the pattern of LN-derived AZs (Ng et al., 2002; Mosca and Luo, 2014). These inhibitory interneurons, unlike PNs, respond transiently to odor stimuli (Nagel et al., 2015). This attribute enables the activities of LNs to signal the onset/offset or concentration fluctuations of odor. The LNs receive diverse synaptic inputs, including excitatory synapses from ORN and PN, and inhibition from other LNs (Yaksi and Wilson, 2010). The LN-to-LN synapses are slow and facilitating (Nagel and Wilson, 2016). Intriguingly, Unc13B is the dominant isoform at these synapses. Knocking down of Unc13B at LN terminals greatly reduced the inhibitory transmission between LN and LN (Fulterer et al., 2018). The A isoform, though it coexists with Unc13B at this synapse type, has only an insignificant effect on synaptic transmission, implying that the B isoform functionality dictates the slow release character at these synapses.

In addition to these electrophysiological findings at different synapses, Ca^2+^ imaging conveyed a similar idea that Unc13A promotes a fast, transient release component, whereas Unc13B contributes to a slow and tonic component at the very same ePN-to-KC synapses in the mushroom body calyx (Pooryasin et al., 2021). Importantly, different Unc13 isoforms promoting distinct SV release components is not a unique phenomenon restricted to *Drosophila* or insects. Indeed, the nematode *C. elegans* also expresses two isoforms of Unc13, namely, a long form, Unc13-L, and a short one, Unc13-S, again differing in their N-termini. Unc13-L harbors a C2A domain, which is missing in the Unc13-S N-terminus. The long form localizes in the close proximity to the calcium entry site of *C. elegans* NMJ, while Unc13-S displays a more diffuse distribution. Unc13-L missing the N-terminal part also displayed diffuse axonal localization, suggesting the precise AZ location of the protein defined by its N-terminus as in *Drosophila*. The long form near the Ca^2+^ source promotes fast vesicular release, while the slow component is apparently mediated by both isoforms (Hu et al., 2013, 2015). Finally, different Munc13 isoforms were also found to mediate distinct components of short-term plasticity in mammals. Synapses employing bMunc13-2 get potentiated, while those relying on Munc13-1 get depressed upon repetitive stimulation (Rosenmund et al., 2002; Zhou et al., 2013; Kawabe et al., 2017). Thus, different Unc13 isoforms, obviously across evolution, endow synapses with different release kinetics and favor different forms of short-term plasticity, acting as different temporal filters of signal transduction (Mukunda and Narayanan, 2017) and contributing to the heterogeneity of information coding.

## Distinct Roles of Unc13A and B in Information Decoding to Steer Olfactory Behavior

Short-term plasticity imposes a filtering function on synaptic information transmission (Fortune and Rose, 2001); therefore, depressed A-component and B-mediated facilitated components probably contribute differently to tuning sensory coding. The *Drosophila* olfactory system generating olfactory smell behavior is a particularly well-studied system. Here, odor information sensed by ORN is further transmitted to two higher processing centers, namely, the mushroom body and the LH. The latter structure mediates innate olfactory behavior, receiving not only the excitatory input via ePNs but also the inhibitory input from iPNs (Tanaka et al., 2012). The iPNs thereby were suggested to act as a high-pass filter of transmitter release from ePNs (Parnas et al., 2013). The ORN-to-PN connections follow a nearly one-to-one manner, yet PNs are more broadly tuned by odor than ORNs owing to signal transmission being carried by two kinetically distinct EPSC components on different time scales and dynamic inhibition from LNs (Kazama and Wilson, 2008; Nagel et al., 2015). Unlike the probably largely random PN-to-KC inputs, olfactory inputs to LH neurons (LHNs) are rather stereotyped and biased to certain overrepresented PN glomeruli. Different morphological types of LHNs exhibit distinct information computation features that depend on their specific connectivity patterns. The LHNs receiving pooled excitatory coactivated PN inputs are broadly tuned to odors. By contrast, neurons combining excitation and inhibition from coactivated PNs are narrowly tuned (Fisek and Wilson, 2014; Jeanne and Wilson, 2015; Jeanne et al., 2018). In this regard, Unc13A at the ePN AZs was found to be required for the proper sensation of odor and their valence (appetitive or aversive). By contrast, the B isoform here seemingly encodes a more generic aversive valence of inputs captured (Pooryasin et al., 2021). In addition, the Unc13B-mediated release component of the iPNs acts as an antagonistic signal to that of ePNs, which can reset the attraction-shifted innate smell responses caused by Unc13B downregulation at ePNs. The behavioral alterations are imputed to the loss of isoform-specific release components rather than developmental deficits or a complete destruction of AZ structure, as experiments employing temporally restricted downregulation of either isoform or interfering with the AZ nano-topology by expression of only the C-terminal fragments that generate ectopic release sites (Reddy-Alla et al., 2017) converged to very similar conclusions.

In another context, Unc13A and Unc13B, as said above, enriched in different mushroom body lobes, are also probably of importance for circuits encoding and storing discrete memory components. Short-term associative aversive olfactory memory was found impaired when either isoform was knocked down in KCs (Bohme et al., 2019; Woitkuhn et al., 2020). The short-term memory deficit caused by Unc13A downregulation could not be restored by re-expressing the C-terminal part of it, which generates release sites at atypical locations as mentioned above (Bohme et al., 2019). Mid-term memory performances were impaired in both Unc13A and B downregulated conditions as well, though to a much lesser extent when knocking down only the B isoform in KCs (Woitkuhn et al., 2020). The mid-term memory is composed of an anesthesia-sensitive and an anesthesia-resistant memory (ARM) component (Quinn and Dudai, 1976). Notably, the anesthesia-sensitive memory component measured 3 h after training was completely abolished in Unc13A knocked-down flies. Meanwhile, ARM performance was deteriorated in the A isoform deletion scenario but remained unaffected in Unc13B knocked-down animals. These distinct memory phases and components rely on different subsets of KCs. The Unc13A-rich γ lobe particularly encodes short-term memory and is recruited during retrieval of the ARM component (Bouzaiane et al., 2015). In addition, the early memory trace after training is found in the α′ and β′ lobes whose AZs are also enriched in Unc13A (Wang et al., 2008). Outputs from these two lobes are required for forming a solid mid-term memory (Krashes et al., 2007) and retrieval of a long-term ARM (Bouzaiane et al., 2015). In contrast, the α and β lobes with a relatively high Unc13B/Unc13A ratio are involved in anesthesia-sensitive memory and a short-term ARM retrieval. Thus, memory form-specific impairment caused by isoform A or B downregulation might, to some extent, be attributable to the stoichiometric differences in the levels of the two isoforms across mushroom body lobes. How exactly Unc13 isoform-specific release components interact and differentially filter KC activity patterns instrumental for forming distinct memory components is an issue now warranting research.

The *Drosophila Unc13* locus has also been linked to behavioral responses to ethanol exposure. At a sedating concentration, ethanol decreased the release of the pre-synaptic vesicle from ORNs elicited by odor presentation, and, surprisingly, this reduction was less pronounced in *Unc13* heterozygous loss-of-function mutants based on measurements using synapto-pHluorin. The heterozygotes were also less sensitive to the sedative effects of ethanol measured by the loss of righting reflex assay (Xu et al., 2018) and, at the same time, showed an increased preference for ethanol in the so-called CAFÉ assay (Das et al., 2013). Ethanol interacts with the C1 domain at a conserved Glu-residue, which is shared by both isoforms (Das et al., 2013). A low concentration of ethanol could already inhibit the interaction between diacylglycerol and the C1 domain *in vitro* (Xu et al., 2018), which lowers the energy barrier for SV fusion and influences the probability of the vesicle release (Basu et al., 2007). However, the behavioral regulation does not seem to be conserved in heterozygous *munc13-1* mice, potentially due to a compensatory effect by other Munc13 isoforms (Wooden et al., 2020). Here again, its accessibility to the genetic analysis makes *Drosophila* an attractive model for investigating the role of synaptic plasticity in ethanol intoxication and addiction.

## Different Roles of Unc13 A and B in the Plastic Remodeling of Active Zones

Memory formation is accompanied and mediated by the synaptic growth and structural remodeling. Are Unc13A and Unc13B, which contribute to varied forms of learning behaviors, involved in this process as well? As has been said, synaptic transmission seems dominantly controlled by Unc13A at CNS fast synapses and the peripheral larval NMJs. Notably, Unc13A, which predicts single AZ release activity by controlling its SV release probability, has just been found to be involved in pre-synaptic homeostatic plasticity. The latter can be triggered at the NMJ of *Drosophila* larvae by the application of a glutamate receptor blocker Philanthotoxin, resulting in a compensatory enhancement of pre-synaptic neurotransmitter release by upregulation of both the SV-release probability at existing release sites and the number of functional release sites (Muller et al., 2012; Lazarevic et al., 2013; Li et al., 2018). When resolved by STED, the number of BRP/Unc13A clusters at a single AZ, probably corresponding to the release sites per AZ, varied greatly, but mostly ranged from two to six clusters. Within minutes after Philanthotoxin treatment, seemingly new BRP/Unc13A nanomodules were incorporated into remodeling AZs, resulting in a right shifting of the distribution of the cluster number per AZ. This rapid remodeling process, which is essential to consolidate release potentiation, is lost in *unc13A* mutants, indicating its indispensable role in the plastic augmentation of SV release. The incorporation of nanomodules depends on the stable and precise location of Unc13A at the AZ via the N-terminus. As has been described above, the Unc13A C-terminal fragments, which are incapable of pre-synaptic homeostatic plasticity, could not recover the short-term memory impairment provoked by the Unc13A downregulation in the memory center of *Drosophila*, linking its plasticity regulation function to memory formation (Bohme et al., 2019).

The early onset of the Unc13B during the AZ assembly may indicate its involvement in development. Synaptic release probabilities at mature NMJs are dominantly defined by Unc13A. Null mutants of *Unc13B* only showed a mild reduction in the response evoked (Bohme et al., 2016). However, Unc13B deletion during the early developmental stage could also result in an altered release probability. A recent study by Ramesh et al. (2021) revealed that the release component meditated by Unc13B at nascent AZs facilitated glutamate receptor incorporation at opposing post-synapses. The knocking down of Unc13B in motoneurons suppressed the GluRIIA accumulation at the NMJ, while gain-of-function *Unc13B* mutation with enhanced glutamate releasement (Lipstein et al., 2017) drastically promoted GluRIIA accumulation (Ramesh et al., 2021). This operation is again partially mediated by its interaction with Syd-1.

## Outlook

*Drosophila* Unc13 isoforms are nano-clustered by distinct AZ scaffolding proteins and thus form release sites that are either tightly or loosely coupled to Ca^2+^ channels. They change the AZ release characteristics, probably as a direct consequence, providing functional diversity to *Drosophila* synapses and obviously beyond. The stoichiometric mixing of Unc13A and B likely evolved to endow synapses with a dynamic range of optimal frequencies for transmission, accordingly, tuning specific activity patterns in support of specific behavioral components. These relations in *Drosophila*, with its unique possibilities for behavioral genetics, as well as in mammals, should be investigated for deepening our understanding of how AZ-based information filtering contributes to information processing and, consequently, behavior.

## Author Contributions

CP wrote the first draft of the manuscript. SS and CP reviewed and edited the manuscript. Both authors contributed to the article and approved the submitted version.

## Funding

This work was supported by the OpenAccess Publication Fund of Freie Universität Berlin. The support of the Deutsche Forschungsgemeinschaft (DFG) (FOR2705 [Project-ID: 365082554], FOR5228 [Project-ID: 447288260], SFB1315 [Project-ID: 327654276], and SI 849/14-1 [Project-ID: 445178831]) was gratefully acknowledged.

## Conflict of Interest

The authors declare that the research was conducted in the absence of any commercial or financial relationships that could be construed as a potential conflict of interest.

## Publisher's Note

All claims expressed in this article are solely those of the authors and do not necessarily represent those of their affiliated organizations, or those of the publisher, the editors and the reviewers. Any product that may be evaluated in this article, or claim that may be made by its manufacturer, is not guaranteed or endorsed by the publisher.
